# Epidemiologic and Genomic Surveillance of *Vibrio cholerae* and Effectiveness of Single-Dose Oral Cholera Vaccine, Democratic Republic of the Congo

**DOI:** 10.3201/eid3102.241777

**Published:** 2025-02

**Authors:** Christine Marie George, Alves Namunesha, Kelly Endres, Willy Felicien, Presence Sanvura, Jean-Claude Bisimwa, Jamie Perin, Justin Bengehya, Jean Claude Kulondwa, Ghislain Maheshe, Cirhuza Cikomola, Lucien Bisimwa, Alain Mwishingo, David A. Sack, Daryl Domman

**Affiliations:** Johns Hopkins Bloomberg School of Public Health, Baltimore, Maryland, USA (C.M. George, K. Endres, J. Perin, D.A. Sack); Université Catholique de Bukavu, Bukavu, Democratic Republic of the Congo (A. Namunesha, W. Felicien, P. Sanvura, J.-C. Bisimwa, G. Maheshe, C. Cikomola, L. Bisimwa, A. Mwishingo); Ministère de la Santé, Bukavu (J. Bengehya, J.-C. Kulondwa); University of New Mexico Health Sciences Center, Albuquerque, New Mexico, USA (D. Domman)

**Keywords:** cholera, *Vibrio cholerae*, epidemiology, surveillance, whole-genome sequencing, Preventative Intervention for Cholera for 7 Days program, PICHA7, vaccines, bacteria, enteric infections, Democratic Republic of the Congo

## Abstract

We conducted 4 years of epidemiologic and genomic surveillance of single-dose effectiveness of a killed whole-cell oral cholera vaccine (kOCV) and *Vibrio cholerae* transmission in the Democratic Republic of the Congo. We enrolled 1,154 patients with diarrhea; 342 of those had culture-confirmed cholera. We performed whole-genome sequencing on clinical and water *V. cholerae* isolates from 200 patient households, which showed annual bimodal peaks of *V. cholerae* clade AFR10e infections. A large clonal cholera outbreak occurred 14 months after a kOCV campaign of >1 million doses, likely because of low (9%) vaccine coverage in informal settlements. Clinical and water isolates collected in the same household were closely related, suggesting person-to-person and water-to-person transmission. Single-dose kOCV vaccine effectiveness 24 months after vaccination was 59.8% (95% CI 19.7%–79.9%), suggesting modest single-dose kOCV protection. kOCV campaigns combined with water, sanitation, and hygiene programs should be used to reduce cholera in disease-endemic settings worldwide.

An estimated 2.9 million cholera cases and 95,000 deaths occur annually in cholera-endemic countries (*1*). The Democratic Republic of the Congo (DRC) has one of the highest rates of cholera in Africa (*2*). In 2017, the Global Task Force on Cholera Control released the document Ending Cholera: A Global Roadmap to 2030 to reduce cholera deaths by 90% in the DRC and eliminate cholera in 20 other countries by 2030 (*3*). To address cholera in transmission hotspots, the task force recommends oral cholera vaccine (OCV) campaigns and case area–targeted water, sanitation, and hygiene (WASH) interventions should be implemented in a ring around cholera cases. However, a global OCV stockpile shortage has necessitated identifying the duration of protection conferred by OCVs to determine the optimal frequency for OCV campaigns (*4*). Most studies on OCV effectiveness have been from India and Bangladesh and have evaluated the effectiveness of 2-dose OCV protection (H. Xu et al., unpub. data, https://doi.org/10.1101/2024.08.13.24311930). Only 4 studies have evaluated single-dose OCV vaccine effectiveness (2 of those for a duration of >24 months), and all except 1 used the Shanchol vaccine, which is no longer produced (H. Xu et al., unpub. data).

Few studies have combined evaluations of OCV effectiveness and genomic surveillance of clinical and environmental *Vibrio cholerae* isolates by using whole-genome sequencing (WGS), which can provide valuable information on how OCV campaigns affect *V. cholerae* transmission dynamics and can identify genetic characteristics of circulating *V. cholerae* strains. WGS is also a valuable tool to link cholera epidemics globally and investigate *V. cholerae* transmission by distinguishing strains on the basis of single-nucleotide polymorphisms (SNPs) (*5*). Genomic data from 45 countries in Africa revealed that the *V. cholerae* seventh pandemic El Tor (7PET) strain was introduced into Africa >15 times since 1970 (*6*). Previous genomic studies (2018–2024) in DRC have found *V. cholerae* 7PET strains belong to clades AFR10d, AFR10e, and ARFR10w (*7*,*8*).

We previously used WGS to analyze water and clinical sources of *V. cholerae* collected from patient households to investigate cholera transmission dynamics in Bangladesh (*9*). We found that 80% of cholera patient households had isolates from water that were closely related to clinical isolates, whereas 20% of households had clinical isolates from infected persons that were more closely related to clinical isolates from other households than to source water isolates in their own household. Those results were consistent with person-to-person and water-to-person *V. cholerae* transmission. Genomic studies to elucidate transmission dynamics of *V. cholerae* infection in cholera patient households in sub-Saharan Africa are lacking; previous studies have been exclusively in South Asia.

We conducted 4 years of epidemiologic and genomic surveillance of *V. cholerae* in eastern DRC to achieve the following aims. First, we evaluated the effectiveness of a single-dose of Euvichol-Plus (EuBiologics, http://eubiologics.com), a killed whole-cell OCV (kOCV), during the 24-month period after a preventive kOCV campaign. Second, we investigated *V. cholerae* transmission dynamics among cholera patients, household members, and water sources by using WGS. Third, we determined the spatiotemporal spread of *V. cholerae* in this cholera-hyperendemic region.

## Methods

### Ethics Approval

We conducted this study in urban Bukavu, South Kivu Province, DRC. We received ethics approval for this study from Johns Hopkins Bloomberg School of Public Health, (Baltimore, Maryland, USA) and Catholic University of Bukavu (Bukavu, DRC). All participants or their guardians provided written informed consent.

### Study Design and Protocol

During March 2020–March 2024, we conducted passive cholera surveillance at 115 healthcare facilities in Bukavu. Patients with diarrhea who were admitted during this time had their feces tested for *V. cholerae* by using bacterial culture. We defined cholera patients as patients with diarrhea who had a *V. cholerae*–positive fecal sample by bacterial culture. We conducted a prospective cohort study of household contacts of cholera patients during December 2021–December 2023 to investigate cholera transmission dynamics within cholera patients’ households. We defined household contacts of cholera patients as those sharing a cooking pot and residing in the same home with the cholera patient for the previous 3 days. We enrolled household contacts in the study within 24 hours of enrolling the corresponding cholera patient. We determined the sample size for the prospective cohort study by using the number of cholera patients who could be screened and who were willing to participate in the cohort study. We visited cholera patient households on days 1, 3, 5, 7, 9, and 11 (visits 1–6) after the household’s index cholera patient was admitted at a health facility to conduct clinical surveillance. For clinical surveillance, we collected a fecal sample from the cholera patient and household contacts during each household visit to test for *V*. *cholerae* by using bacterial culture. We conducted an unannounced spot check at each timepoint to collect a sample of the household’s water source and stored drinking water to test for *V*. *cholerae* by bacterial culture.

During December 28, 2021–January 2, 2022, and March 31–April 4, 2022, the DRC Ministry of Health delivered 1.04 million doses of Euvichol-Plus kOCV in Bukavu as a preventive OCV campaign. Vaccines were delivered through a combination of door-to-door visits and designated healthcare facilities. We assessed OCV vaccination status for patients with diarrhea and their household members through self or caregiver reporting during the time of patient treatment in the healthcare facility or during a home visit conducted the same or the following day. Study research officers administered a structured questionnaire, which obtained information on OCV administration and the date and number of doses. An OCV vaccine card was shown, along with a photo of the person consuming OCV. We defined informal settlements as areas where no household connections to piped water existed.

### Laboratory Analyses

All whole fecal samples were brought to the Preventative Intervention for Cholera for 7 Days program Enteric Disease Microbiology Laboratory in Bukavu within 3 hours of the sample being produced, and water samples were brought to the laboratory within 3 hours of collection for *V*. *cholerae* analysis by bacterial culture as previously described (*10*). We preserved isolated bacteria as stabs in nutrient agar or on Whatman filter paper to preserve bacterial DNA.

### WGS

We extracted DNA from isolates preserved as agar stabs by using the ZymoBIOMICS DNA Miniprep Kit (Zymo Research, https://www.zymoresearch.com) and extracted genomic bacterial DNA from filter paper by using published Chelex methods (*11*). The SeqCenter (https://www.seqcenter.com) and University of New Mexico Health Sciences Center performed WGS. We processed short reads by using the Bactopia pipeline (*12*) and SPAdes version 3.10.0 (*13*) and annotated by using Prokka version 1.520 (*14*). We performed genome completeness estimates and checks for contamination by using CheckM version 1.0.722 and Kraken version 0.10.6 (*15*,*16*). We deposited all next-generation sequencing data from this study under the National Center for Biotechnology Information BioProject database (https://www.ncbi.nlm.nih.gov/bioproject; no. PRJNA1210607).

### Genomic and Phylogenetic Analyses

We mapped paired-end reads for 255 7PET isolates and compared variants with the *V. cholerae* O1 El Tor reference genome N16961 (GenBank accession nos. LT907989 and LT907990) by using snippy version 4.6.0 (https://github.com/tseemann/snippy) via the Bactopia pipeline (*12*) to generate a reference-based alignment containing 329 variable SNP sites. All 255 isolates mapped with >90% of the reference genome; thus, we further analyzed all 255 isolates. We generated a pairwise SNP matrix for the 255 7PET isolates by using pairsnp (https://github.com/gtonkinhill/pairsnp). We used ARIBA version 2.14.7 (https://github.com/sanger-pathogens/ariba) to determine mutations in the *wbeT* gene by comparing sequences with a reference wild-type Ogawa *wbeT* gene from the European Nucleotide Archive database (https://www.ebi.ac.uk/ena; accession no. AEN80191.1)

We compared 7PET isolates from this study with 1,418 globally representative 7PET strains to assess phylogeographic relatedness (*17*). We used the 12,561 variable site (SNPs) alignment to build a maximum-likelihood phylogeny; we used IQ-Tree version 1.6.12 and the general time reversible substitution model with the gamma distribution to model site heterogeneity with 10,000 ultrafast bootstraps and used 10,000 bootstraps for the Shimodaira-Hasegawa–like approximate-likelihood ratio tests (*18*). We visualized phylogenies by using ggtree version 1.6.11 (*19*) and rooted the trees by using the pre-seventh pandemic *V. cholerae* strain M66.

### Local Transmission Analyses

We gained insights into local transmission dynamics from a 329 SNP reference-based alignment of the 255 7PET isolates. We built a maximum-likelihood tree by using IQ-Tree and the models and bootstraps as described previously. We visualized phylogenies by using Microreact (*20*). We created minimum spanning trees by using GrapeTree version 1.5.0 (*21*) and visualized geospatial data for cases by using Python geopandas (https://github.com/geopandas/geopandas) and contextily (https://github.com/geopandas/contextily) packages.

### Statistical Analysis

We assessed single-dose Euvichol-Plus kOCV effectiveness during the 24-month period after vaccination by using a test-negative design among patients with diarrhea. We used logistic regression models with cholera infection as the outcome (defined as a positive *V. cholerae* bacterial culture) and kOCV vaccination status (whether patients with diarrhea reported receiving 1 dose of kOCV during the kOCV campaign period) as the predictor. In this model, the odds ratio was the odds of single-dose kOCV vaccination effectiveness in the cholera-positive patients with diarrhea compared with controls (non–cholera patients with diarrhea). We calculated vaccine effectiveness by using the equation (1 – OR) × 100%, and we excluded persons who reported receiving 2 doses of kOCV from the analysis. We performed analyses by using SAS version 9.4 (SAS Institute Inc., https://www.sas.com). We performed permutation tests by using R (The R Project for Statistical Computing, https://www.r-project.org) and Python to analyze pairwise comparisons of genomic data. For pairwise comparisons, we compared SNP counts for each strain with those from the *V. cholerae* reference strain N16961.

## Results

### Epidemiology

**Figure 1:**
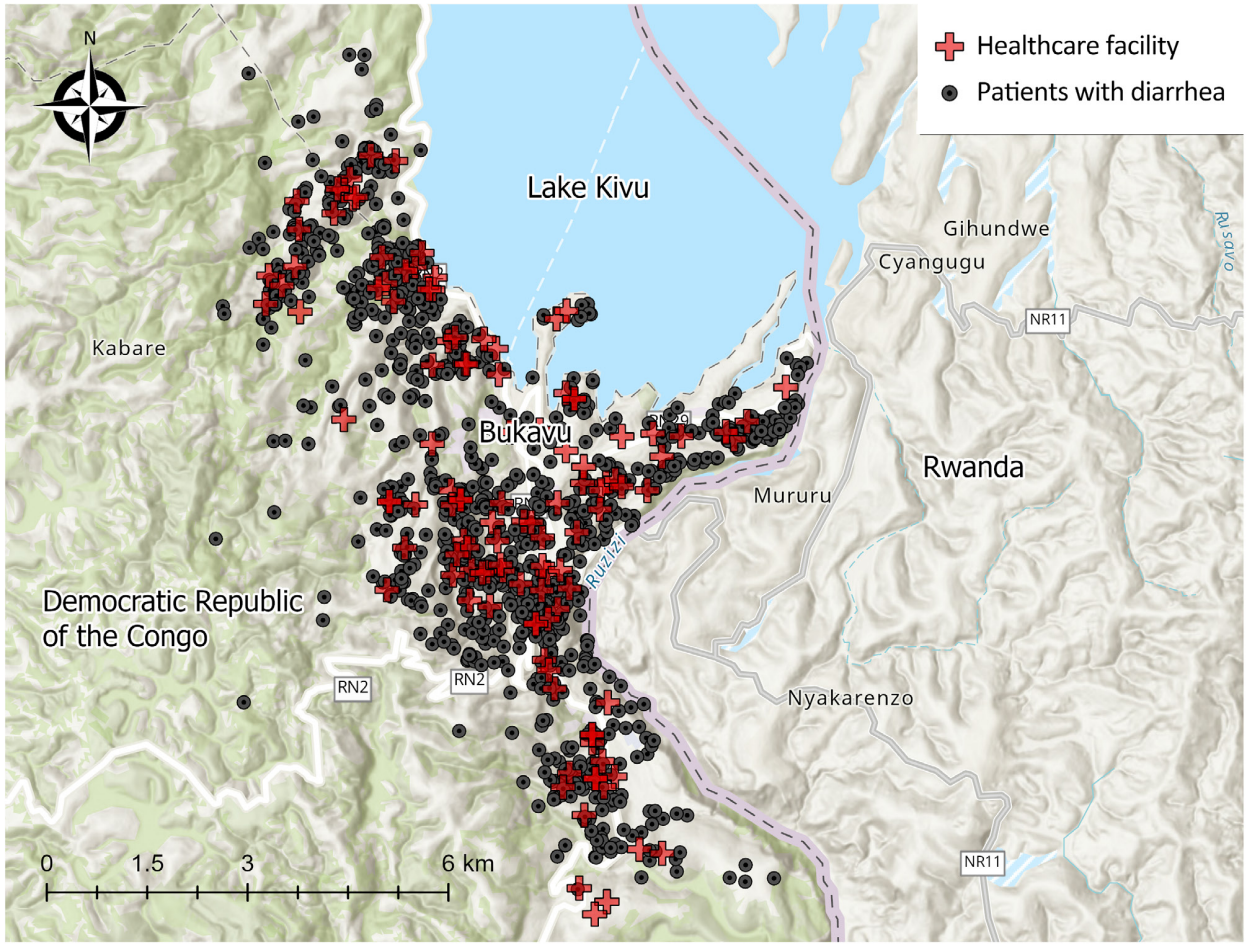
Location of patients with diarrhea and the corresponding healthcare facilities where they sought treatment in a surveillance study of *Vibrio cholerae* and effectiveness of single-dose oral cholera vaccine, Democratic Republic of the Congo, 2020–2024. A total of 115 healthcare facilities and 1,098 households of patients with diarrhea are shown; 56 patient households had missing Global Positioning System coordinates.

During March 2020–March 2024, we identified *V. cholerae* in fecal samples from 342 (30%) of 1,154 patients with diarrhea via bacterial culture (Appendix Figure 1). We mapped the 115 healthcare facilities and households of patients with diarrhea surveilled in this study (Figure 1). We observed an annual bimodal peak of cholera during the dry (June–August) and rainy (September–January) seasons in 2020 and 2023 (Appendix Figure 2). In December 2021, a kOCV campaign distributed 1.04 million doses of Euvichol-Plus within Bukavu (study surveillance site). After that kOCV campaign, sporadic cholera patients were observed through January 2023. Then, ≈14 months after the kOCV campaign (June–November 2023), a large cholera outbreak occurred in this same area. Nine percent (309/3,395) of persons residing in informal settlements within the study area reported receiving >1 kOCV dose during December 2021–April 2022. During the 4-year surveillance period, stored and source water samples were collected from 178 cholera patient households; 9% (16/173) of households had *V. cholerae*–positive stored water samples and 6% (9/157) of households had positive source water samples. A total of 29 water samples were *V. cholerae* positive over the study period (Appendix Figure 3). During October 2021–November 2022 (including the 12-month period after the kOCV campaign was initiated), no *V. cholerae* was detected in drinking water samples, despite the presence of culture-confirmed cholera patients.

**Figure 2:**
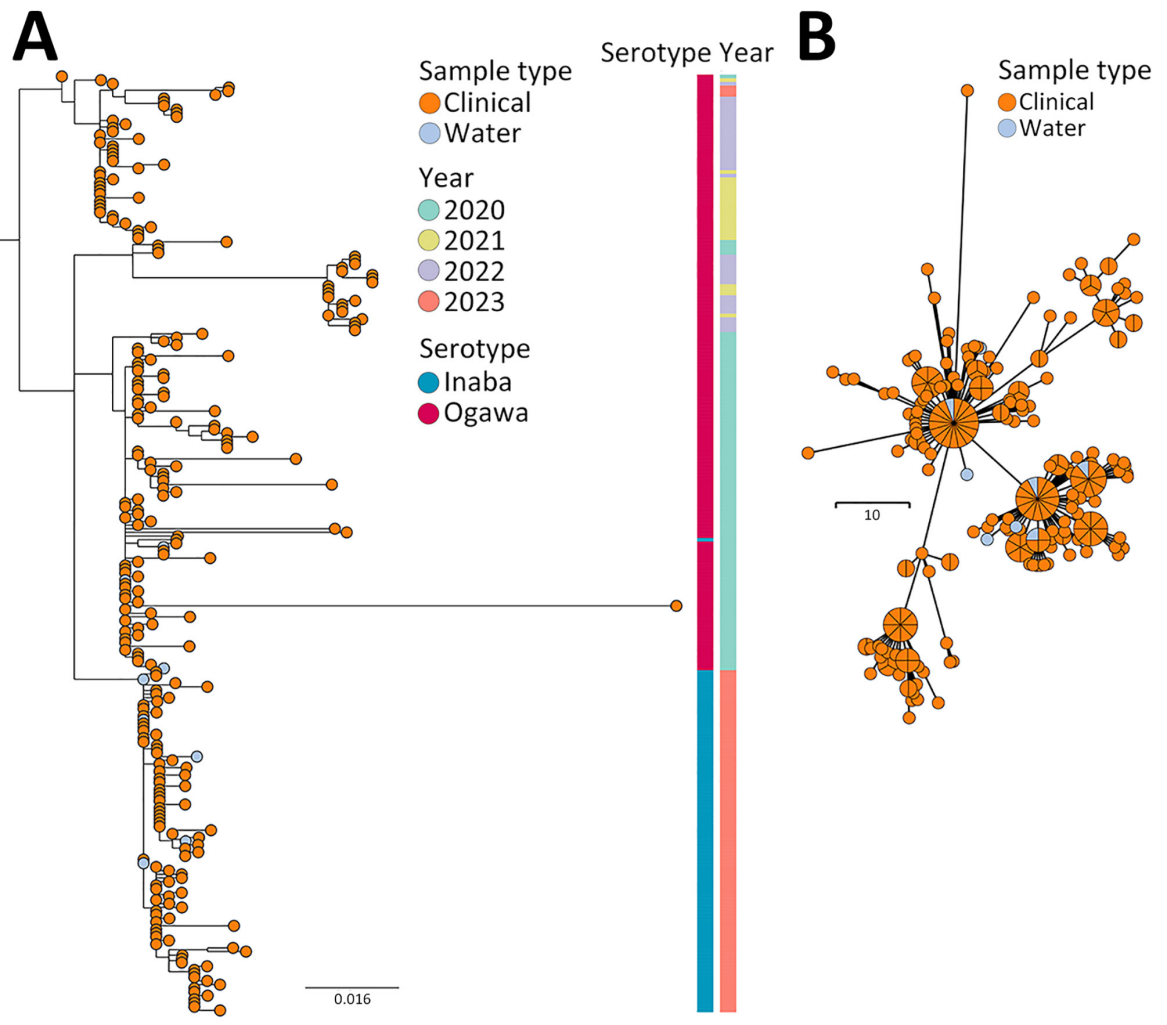
Phylogenetic analysis of *Vibrio cholerae* seventh pandemic El Tor (7PET) isolates collected during study of *V*. *cholerae* transmission and effectiveness of single-dose oral cholera vaccine, Democratic Republic of the Congo. A) Maximum-likelihood tree of 255 7PET *V. cholerae* genomes sampled in Bukavu during 2020–2023. Node colors indicate sample type. Associated colored metadata indicate sampling year and inferred serotypes according to genome analysis. Scale bar indicates nucleotide substitutions per site. B) Minimum spanning tree of 255 7PET *V. cholerae* genomes sampled in Bukavu during 2020–2023. Node colors indicate sample type. Isolates with 0 single-nucleotide polymorphism differences between each other are collapsed into single node. Node sizes are scaled according to the number of samples. Scale bar indicates single-nucleotide polymorphisms.

**Figure 3:**
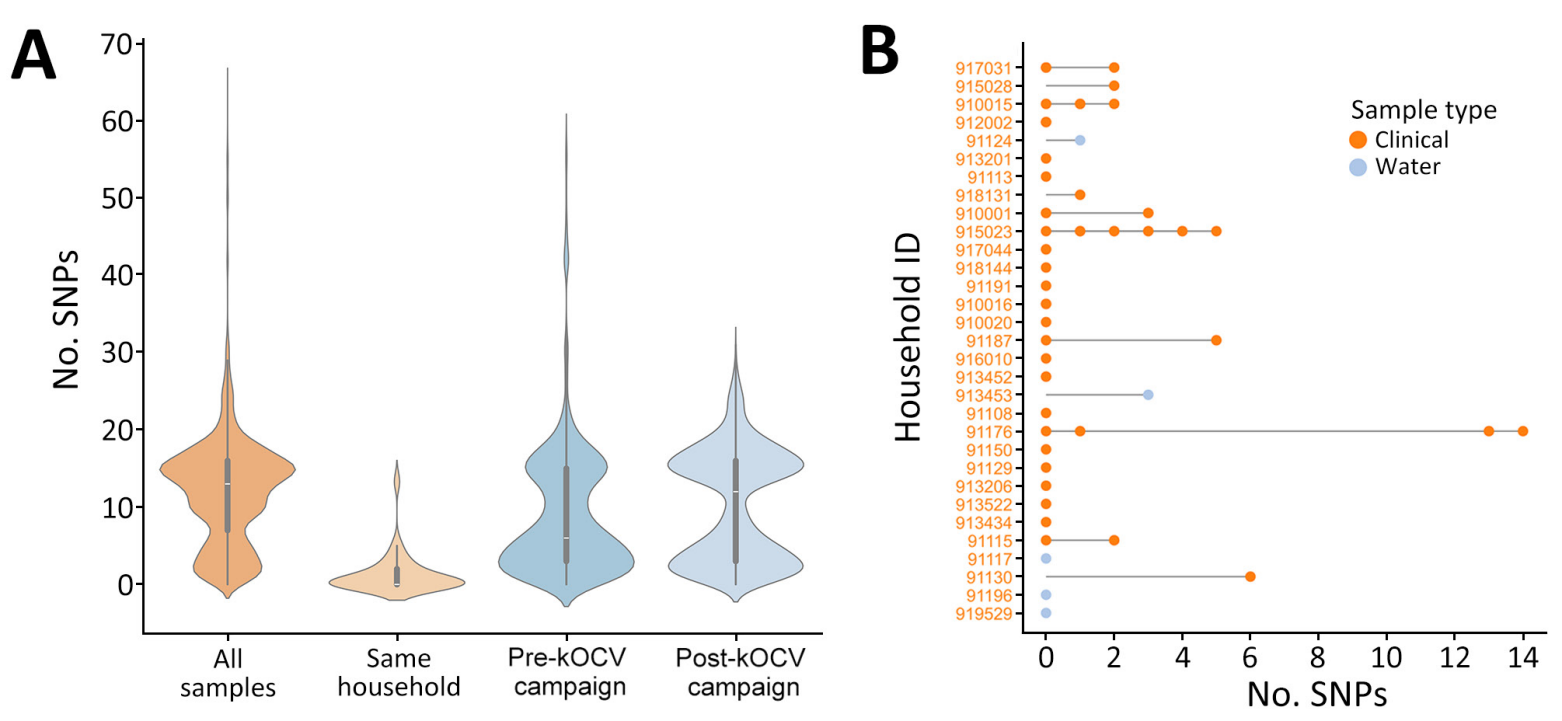
Pairwise comparisons of SNPs in surveillance study of *Vibrio cholerae* and effectiveness of single-dose killed oral cholera vaccine (kOCV), Democratic Republic of the Congo. A) Violin plot showing distribution of pairwise SNP differences from all isolates collected during the study, those from the same household, and those pre-kOCV and post-kOCV campaign. Density curves indicate frequency of data points. Inside each density plot, horizontal white lines within boxes indicate medians; box tops and bottoms indicate upper (third) and lower (first) quartiles; and whiskers indicate minimum and maximum values. Number of pairwise comparisons for each category is as follows: all samples, n = 32,385; same household, n = 99; pre-kOCV campaign, n = 6,205; and post-kOCV campaign, n = 10,296. B) SNP differences per household. Pairwise SNP differences are relative to the first sample in the household. Node colors indicate sample type; stems connect samples to their household. ID, identification; SNP, single-nucleotide polymorphism.

### OCV Vaccine Effectiveness

Surveillance healthcare facilities admitted 750 patients with diarrhea during the 24-month period after the kOCV campaign (December 2021–December 2023). We recorded demographic characteristics for kOCV vaccinated and unvaccinated persons (Table). During the 3 nights before hospitalization, 94% (708/750) of patients with diarrhea reported residing in their current residences; 12% (93/748) had running water inside their home. Twelve percent (93/750) (15 cholera patients and 78 non–cholera patients with diarrhea) of patients reported receiving >1 dose of kOCV during December 2021–April 2022; only 2% (14/750) of patients reported receiving 2 kOCV doses, and 13% (12/93) of kOCV-vaccinated patients showed a vaccination card. During this period, 208 (193 unvaccinated and 15 kOCV vaccinated) patients had cholera; 531 patients with diarrhea were >1 year of age. The unadjusted single-dose kOCV vaccine effectiveness in the first 24 months after vaccination was 59.8% (95% CI 19.7%–79.9%) for persons >1 year of age and, after adjustment for age (continuous variable), was 58.7% (95% CI 17.3%–79.4%). We also calculated the single-dose kOCV vaccine effectiveness according to time interval (first 12-month and second 12-month period after vaccination) for persons >1 year of age (Appendix Table). We excluded 10 patients with diarrhea from this analysis because they received 2 doses of kOCV.

**Table:** Demographic characteristics of patients with diarrhea in study of effectiveness of single-dose oral cholera vaccine, Democratic Republic of the Congo*

Characteristics	All patients with diarrhea, n = 750	Vaccination status		p value
		Received kOCV, n = 93	No kOCV, n = 657	
Age, y				
Median +SD (min–max)	5 +19 (0–82)	2 +15 (0–63)	7 +19 (0–82)	
0–1	217/750 (29)	30/93 (32)	187/657 (28)	0.527
1–4	148/750 (20)	28/93 (30)	120/657 (18)	0.011
5–14	96/750 (13)	11/93 (12)	85/657 (13)	0.893
>14	289/750 (39)	24/93 (26)	265/657 (40)	0.009
Patient sex				
F	368/750 (49)	45/93 (48)	323/657 (49)	0.977
M	382/750 (51)	48/93 (52)	334/657 (51)	
Running water inside home	93/748 (12)	12/93 (13)	81/655 (12)	1.000
Working mobile phone in home	565/663 (85)	78/85 (92)	487/587 (83)	0.097
Resided in home, y, median +SD (min–max)	7 +3 (1–23)	7 +2 (3–16)	7 +3 (1–23)	0.098
Formal education,† y, median +SD (min–max)	9 +5 (0–23)	7 +5 (0–15)	9 +5 (0–23)	0.028

*Values are no. patients in each group/total no. patients analyzed (%) except as indicated. p values compare vaccination status. kOCV, killed whole-cell oral cholera vaccine. †For those patients >18 years of age.

### Genomic Analyses

We sequenced 255 *V. cholerae* 7PET genomes from samples collected within the study area. Of those genomes, 247 were clinical isolates from the fecal samples of 243 persons residing in 200 households (198 patients and 45 household contacts), and 8 were from water samples. The WGS analysis included 75% (255/342) of cholera patient households during the surveillance period, and all available isolates were sequenced. To investigate the genetic relatedness of the *V. cholerae* isolates, we analyzed the pairwise SNP differences across 255 genomes. Of the 200 households in the study, 31 households were represented by >1 sample, 26 households had >1 study participant with samples, and 5 households had both clinical and water *V. cholerae* isolates. Maximum-likelihood phylogenic analyses of the 255 7PET *V. cholerae* genomes indicated the *V. cholerae* isolated from both clinical and water samples were closely related, and limited genetic divergence occurred over the study period (Figure 2, panels A, B). Among all isolates, the minimum number of SNP differences was 0 and the maximum number was 65 (median 13) (Figure 3, panel A). For isolates from the same person, the range was 0–1 SNP and median 0 SNPs. For isolates from the same household (isolated from both clinical and water samples), the pairwise differences range was 0–14 SNPs (median 0) (Figure 3, panel B). We found a significant difference in the median number of SNPs among isolates from different households compared with those from the same household (p<0.0001 by Mann-Whitney U test). In the 3 of 5 households with both clinical and water *V. cholerae* isolates, the isolates had 0 SNP differences among them; the other 2 households had 1–3 SNP differences between the clinical and water samples. This result provides evidence that the same strain causing infections in household members was also detected in the household water. However, we cannot determine whether the water was the source of infection or whether the water was contaminated after an infection occurred in a household member. The median difference in the number of SNPs was significantly lower for isolates from cholera patients before the kOCV vaccine campaign (median 6 [range 0–58]) compared with the number of SNPs after the kOCV vaccine campaign (median 12 [range 0–31]) (p<0.0001 by Mann-Whitney U test).

**Figure 4:**
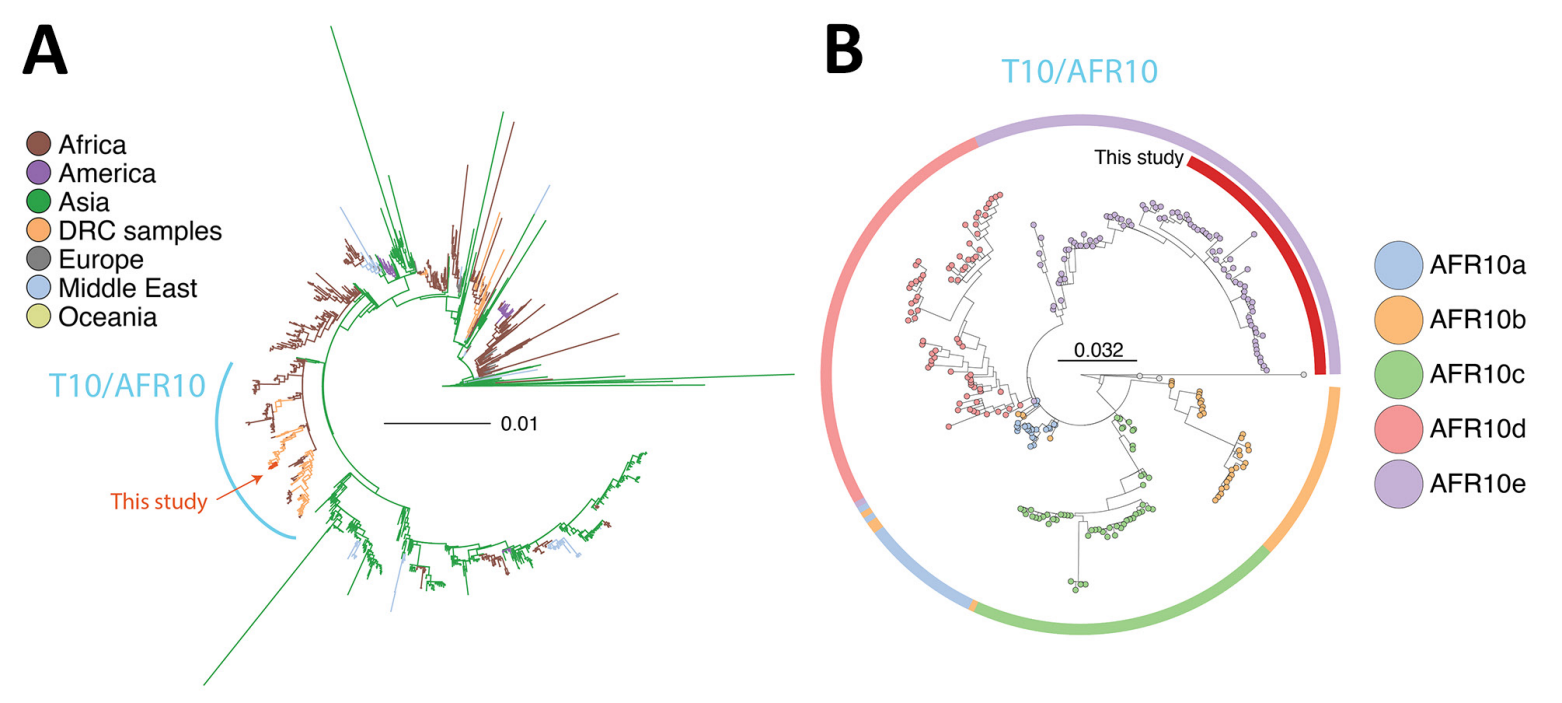
Phylogenetic analysis of *Vibrio cholerae* strains in study of *V. cholerae* transmission and effectiveness of single-dose killed oral cholera vaccine, DRC. Maximum-likelihood phylogenetic trees were prepared to compare *V. cholerae* seventh pandemic El Tor (7PET) isolates. A) Globally representative phylogeny of 1,428 7PET strains; 5 representative isolates from this study (red) were placed within the larger context of those 7PET strains. Tree was rooted on the A6 strain. Branch colors indicate geographic origin of the strain. B) Phylogeny of the T10/AFR10 lineage of *V. cholerae*. Colors indicate different *V. cholerae* lineages. Representative isolates from this study (n = 46; red) were placed within the context of 221 T10/AFR10 lineage strains. Tree was rooted on the reference strain N16961. Scale bars indicate nucleotide substitutions per site. DRC, Democratic Republic of the Congo.

To determine how the 255 *V. cholerae* isolates from this study fit within the larger diversity of 7PET strains, we contextualized those genomes within a global collection of 1,422 additional 7PET genomes (Figure 4) (*17*). Phylogenetic analysis placed the 255 isolates within the T10 lineage of the AFR10e clade. Our isolates, sampled during 2020–2023, were similar to other recent samples (2015–2020) from DRC in the same region of South Kivu (*8*). Genomic analysis of the *wbeT* gene indicated that samples from 2020–2022 had intact wild-type *wbeT* genes, which indicates an Ogawa serotype; we observed 1 exception for a sample from 2020 with a frameshift mutation (N165fs), which would likely result in an Inaba serotype. However, all samples from 2023 after the OCV campaign appear to have a fragmented *wbeT* gene marked by insertion of mobile elements, likely conferring an Inaba serotype. In line with other AFR10e strains, our isolates harbored mutations in *gyrA* (S83I) and *parC* (S85L) genes, which have been proposed to reduce susceptibility to fluoroquinolones (*8*).

## Discussion

In the urban cholera-endemic setting in DRC where we conducted our study, annual bimodal peaks of *V. cholerae* clade AFR10e infections corresponded with the dry and rainy seasons. One third of patients with diarrhea attending 115 surveillance healthcare facilities were confirmed to have cholera by bacterial culture; 9% of stored water and 6% of source water samples from cholera patient households contained *V. cholerae*. This finding suggests that both source water and contamination of stored water might be potential transmission routes for *V. cholerae* infections in cholera patient households. A large clonal cholera outbreak occurred 14 months after >1 million doses of Euvichol-Plus kOCV vaccines were distributed in the same area. This large outbreak occurred despite this vaccine campaign, possibly because of the low (9%) kOCV coverage within informal settlements in Bukavu, which are often hotspots for cholera because of limited access to improved drinking water sources, sanitation options, and poor hygienic conditions (*22*). No water samples were positive for *V. cholerae* during the 12-month period after the kOCV campaign was initiated, despite the presence of cholera patients. Future studies should investigate the effect of kOCV campaigns on *V. cholerae* persistence in the environment.

Using a test-negative design, we found that a single dose of Euvichol-Plus provided modest protection against medically attended cholera during the 24 months after vaccination, consistent with findings from other studies (H. Xu et al., unpub. data). This test-negative design to evaluate kOCV effectiveness could be incorporated into existing cholera surveillance activities globally and could be integrated as part of kOCV vaccine campaign rollouts. Our findings suggest that, with increased vaccination coverage in informal settlements, single-dose kOCV campaigns are a promising approach to deliver vaccines along with WASH programs to reduce cholera in the DRC.

Our genomic findings highlight several points. First, we did not detect any samples that grouped in the *V. cholerae* clade AFR10d, a finding that seems to further corroborate that clade AFR10e replaced AFR10d around 2018 in this region (*23*). Second, during the subsequent cholera outbreak after the large kOCV campaign, we observed a change in *V. cholerae* serotype from Ogawa to Inaba, which was likely related to a fragmented *wbeT* gene marked by insertion of mobile elements. This finding is consistent with another study that observed a serotype switch after an OCV vaccination campaign (*24*). In addition, we observed an increase in the number of SNP differences among *V. cholerae* isolates collected after the kOCV campaign, compared with those collected before the campaign. However, both our findings and those from the previous study are observational, and causality cannot be inferred. Third, this study provided evidence that household source water contained the same isolates that caused cholera infections among household members. This result combined with the finding that clinical isolates in the same household were more closely related than isolates from different households (even when no *V. cholerae* was found in household drinking water sources) suggests a combination of person-to-person and water-to-person cholera transmission. Our findings suggest it is critical for WASH programs to place emphasis on both chlorination of household water and handwashing with soap to prevent cholera transmission.

The first strength of our study is the 4-year duration of epidemiologic and genomic surveillance of cholera patients and their water sources before and after a kOCV campaign. Previous longitudinal studies including both clinical and environmental surveillance have been almost exclusively performed in South Asia. Second, we evaluated the effectiveness of a single-dose of Euvichol-Plus over a 24-month period, building on previous studies that focused mostly on 2-doses of kOCV and evaluated the Shanchol kOCV, which is no longer produced. One study evaluated the single-dose kOCV effectiveness of Euvichol-Plus for a period of >24 months in DRC and found 54% effectiveness 12–17 months after vaccination, similar to our study (*25*). In addition, the test-negative design for kOCV effectiveness builds on observational studies using community controls by reducing the likelihood of differences in care-seeking behavior between *V. cholerae*–infected persons and controls, which can introduce substantial bias in observational studies of vaccine effectiveness. Finally, the inclusion of the genomics data enabled us to investigate how water and clinical *V. cholerae* strains evolved over time before and after a kOCV campaign and to investigate *V. cholerae* transmission dynamics in cholera patient households.

The first limitation of our study is that our surveillance focused only on an urban setting; therefore, we cannot generalize our findings to rural settings. Additional data on single-dose kOCV vaccine effectiveness is needed in rural settings globally. Second, as with all observational study designs, misclassification of a patient’s vaccination status and cholera infection outcome is possible. Also, the vaccine campaign in this study was a preventive campaign in an area identified as a hotspot; however, because of a global kOCV vaccine shortage, all cholera vaccine campaigns are currently reactive and respond to outbreaks. Future studies are needed to evaluate single-dose kOCV effectiveness during reactive kOCV campaigns worldwide.

In conclusion, we observed annual bimodal peaks of *V. cholerae* clade AFR10e in an urban cholera-endemic area in eastern DRC. One third of patients with diarrhea admitted to surveillance healthcare facilities were confirmed to have cholera by bacterial culture. A large clonal cholera outbreak occurred 14 months after a single dose kOCV campaign, and the severity of the outbreak might have been related to the low vaccine coverage in informal settlements. Genomic analyses suggest that both person-to-person and water-to-person transmission occurred. A single-dose of Euvichol-Plus kOCV provided modest protection against medically attended cholera during the 24 months after vaccination. Our findings indicate that single-dose kOCV campaigns in combination with WASH programs should be used to reduce cholera disease in cholera-endemic settings worldwide.

Appendix Additional information for epidemiologic and genomic surveillance of *Vibrio cholerae* and effectiveness of single-dose oral cholera vaccine, Democratic Republic of the Congo.
