# Supplementary material for: Epidemiologic and Genomic Surveillance of Vibrio cholerae and Effectiveness of Single-Dose Oral Cholera Vaccine, Democratic Republic of the Congo
Source: Emerg Infect Dis. 2025 Feb;31(2):288–97. doi: 10.3201/eid3102.241777 (PMC11845162; doi:10.3201/eid3102.241777)
Supplement: Appendix — Additional information for epidemiologic and genomic surveillance of Vibrio cholerae and effectiveness of single-dose oral cholera vaccine, Democratic Republic of the Congo. [file 24-1777-Techapp-s1.pdf]

Article DOI: https://doi.org/10.3201/eid3102.241777

EID cannot ensure accessibility for supplementary materials supplied by authors. Readers who have difficulty accessing supplementary content should contact the authors for assistance.

Epidemiologic and Genomic Surveillance of *Vibrio cholerae* and Effectiveness of Single-Dose Oral Cholera Vaccine, Democratic Republic of the Congo

Appendix

Appendix Table. Single-dose kOCV vaccine effectiveness by time interval for individuals >1 year of age*

Time interval, mo	Non–OCV vaccinated patients with diarrhea†		OCV vaccinated patients with diarrhea†		Unadjusted effectiveness OR %‡ (95% CI)	Adjusted effectiveness OR %‡ (95% CI)
	Noncholera	Cholera	Noncholera	Cholera		
0–12	152	18	27	2	62.6 (–185.2 to 86.3)	61.8 (–185.2 to 86.5)
12–24	127	173	13	9	50.8 (–22.6 to 78.9)	48.7 (–18.1 to 79.9)

*Logistic regression with cholera as outcome and OCV status as a predictor. Adjusted analysis was adjusted for continuous age. Participants <1 year of age and participants with 2 reported doses of OCV were excluded from time interval analysis. Participants with 2 reported doses of OCV were excluded from age analysis. OCV, oral cholera vaccine; OR, odds ratio.
†Vaccinated or unvaccinated patients who had cholera or did not have cholera according to bacterial culture.
‡Vaccine effectiveness was calculated according to the equation (1 – OR) ´ 100%.

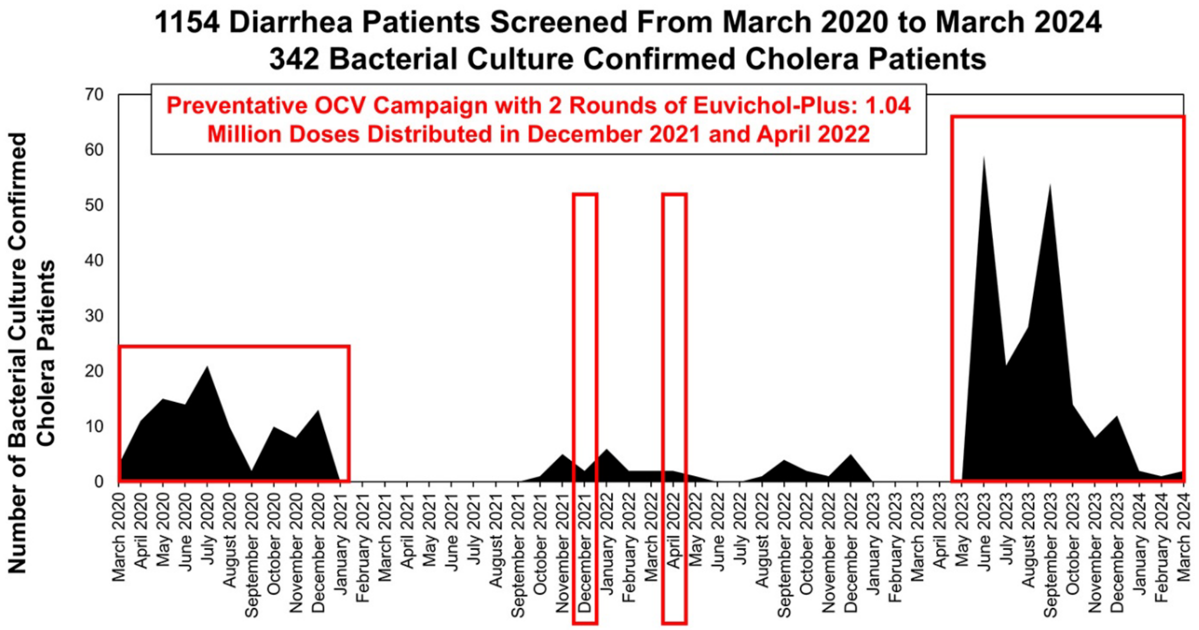

Appendix Figure 1. Four-year epidemiologic surveillance of bacterial culture–confirmed cholera patients from 115 healthcare facilities in Bukavu, South Kivu Province, the Democratic Republic of the Congo, March 2020–March 2024. A total of 1,154 patients with diarrhea were screened at 115 healthcare

facilities, and 342 cholera patients were confirmed by using bacterial culture. Fecal and household water samples were collected for testing. A preventive killed whole-cell oral cholera vaccine campaign was conducted in December 2021 and April 2022.

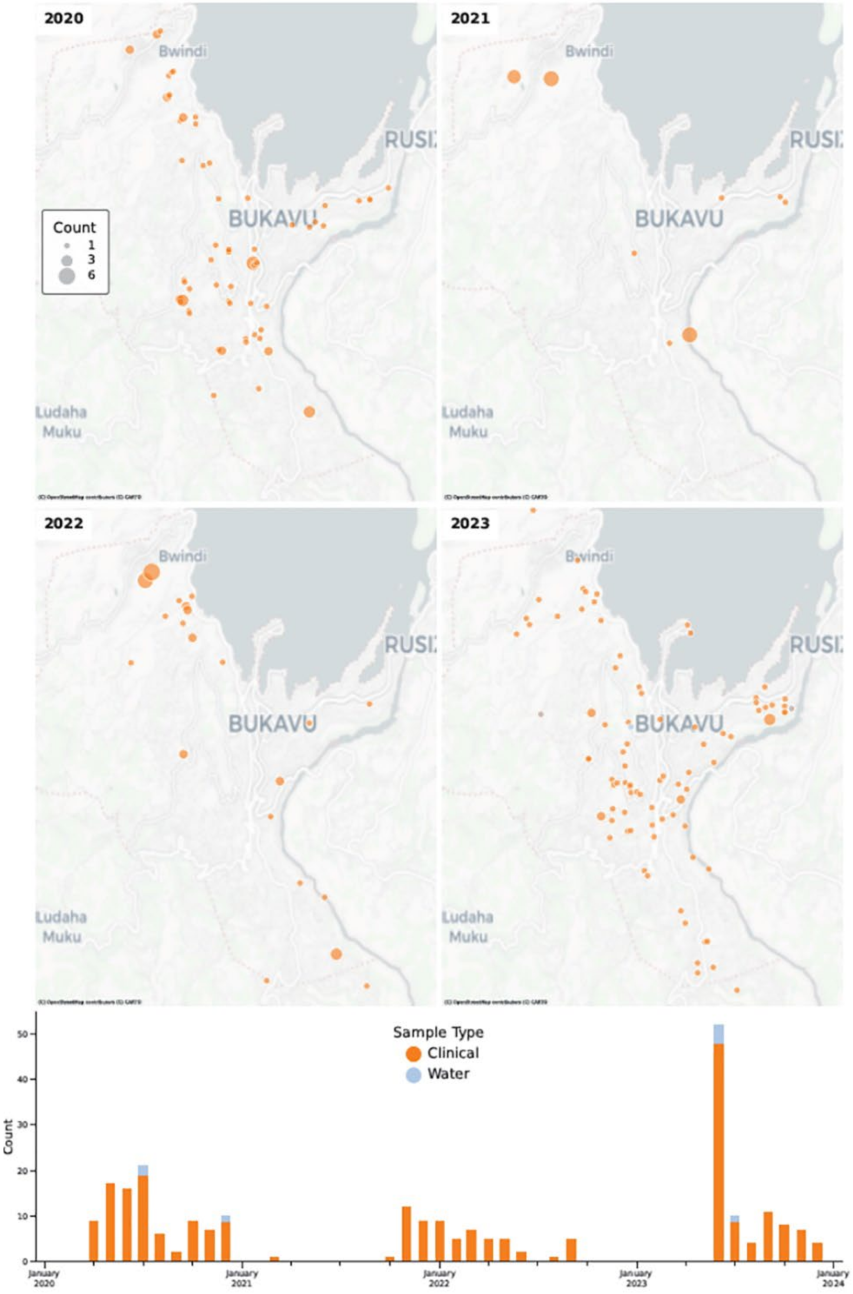

Appendix Figure 2. Locations and counts of cholera patient households in Bukavu, the Democratic Republic of the Congo during 2020–2023. Maps at top indicate spatial distribution of *Vibrio cholerae* isolates and sample type per year. Bottom graph indicates number of samples and sample type per month.

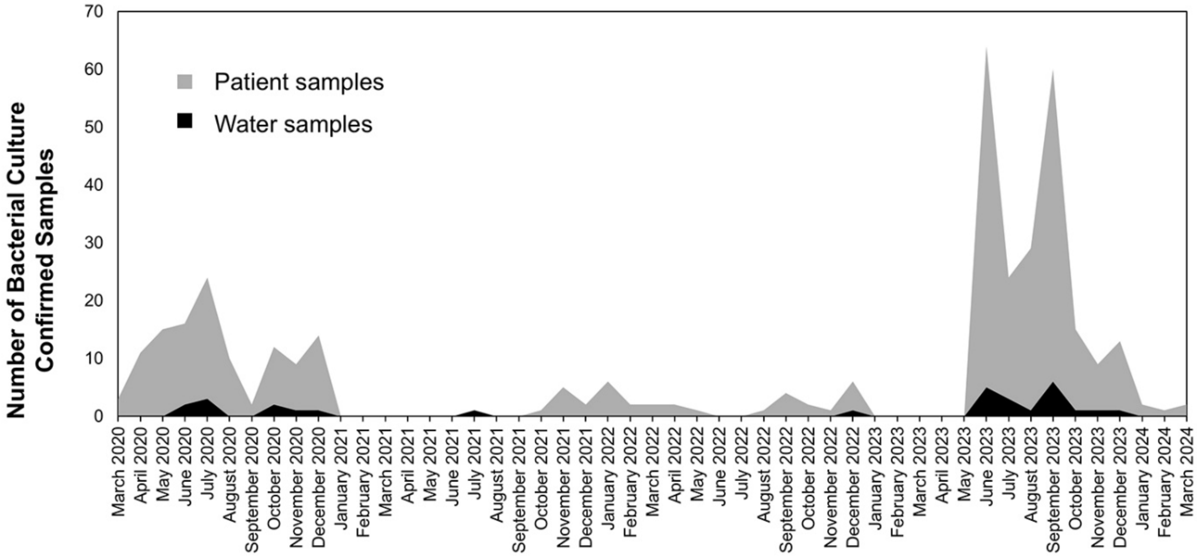

Appendix Figure 3. Four-year epidemiologic surveillance of *Vibrio cholerae* bacterial culture–confirmed cholera patients and water samples from Bukavu, South Kivu Province, the Democratic Republic of the Congo, March 2020–March 2024. A total of 1,154 patients with diarrhea were screened at 115 healthcare facilities and 342 cholera patients were confirmed by bacterial culture; 29 water samples were positive for *Vibrio cholerae* (9 source and 20 stored water samples).
